# ID 106 - Impacto Econômico da Leishmaniose Tegumentar na Perspectiva dos Pacientes: uma análise em um serviço de referência em Minas Gerais, Brasil

**DOI:** 10.5327/2237-9622.2025.v34s1.106

**Published:** 2025-11-25

**Authors:** Sarah Nascimento Silva, Endi Lanza Galvão, Janaína de Pina Carvalho, Mayra Soares Moreira, Tália Santana Machado de Assis, Glaucia Cota

**Keywords:** gastos em saúde, gasto catastrófico em saúde, leishmaniose cutânea, preferência do paciente

## Abstract

**Introdução:**

A leishmaniose tegumentar (LT) é uma doença negligenciada e endêmica em diversos estados do Brasil. As feridas causadas pela doença podem comprometer o desenvolvimento de tarefas e a rotina diária, impactando na qualidade de vida e perda de produtividade do indivíduo. Mesmo no âmbito de um sistema público de saúde universal, os custos para o paciente gerados diretamente pelo tratamento da LT podem ser significativos, além dos gastos indiretos, representados pelas despesas não médicas e perda de receita que os indivíduos sob tratamento vivenciam. O objetivo do trabalho foi identificar os custos diretos e indiretos durante o tratamento da leishmaniose tegumentar sob a perspectiva dos pacientes.

**Método:**

Estudo transversal conduzido no Instituto René Rachou/Fiocruz entre abril de 2022 e abril de 2023, incluindo pacientes com diagnóstico confirmado de LT. Os participantes foram entrevistados durante o período de tratamento para levantamento dos custos diretos médicos, não médicos e custos indiretos associados ao tratamento. Os custos diretos foram estimados pela abordagem de microcusteio e os indiretos pelo método de capital humano. Foram realizadas análises descritivas e testes de hipóteses para associações entre custos e variáveis sociodemográficas e clínicas, com nível de significância de 5%. O estudo recebeu aprovação ética (CAAE 28929220.0.0000.5091).

**Resultados:**

A pesquisa envolveu 68 pacientes com LT, 77,9% do sexo masculino e idade média de 53 anos. A forma cutânea da doença foi a mais comum (76,4%) e a miltefosina foi o tratamento mais utilizado (35,3%). A maioria dos pacientes tinha atividade remunerada ou recebia benefícios do governo, e a renda mensal média foi R$ 2.424,00. Os custos médios por ciclo de tratamento foram de R$ 568,19, sendo o percentual de gasto maior com transporte, alimentação e exames. Gastos catastróficos foram observados em 41,9% das famílias, significativamente associados a custos diretos, infecção bacteriana e fatores sociodemográficos.

**Conclusão:**

Este estudo confirma que o tratamento para LT, embora disponível no SUS, gera gastos diretos e indiretos significativos para os pacientes. Os gastos mais vultosos estão relacionados com as despesas com deslocamento e permanência fora do domicílio, sugerindo um modelo de assistência à saúde centralizado e não disponível no local de residência. A associação da perda de produtividade com terapias que exigem internação hospitalar evidencia a necessidade de uma abordagem individualizada na escolha do tratamento da LT, que considere não apenas os aspectos clínicos da doença, mas também o impacto socioeconômico sobre os pacientes e suas preferências.

